# Links between DNA Replication, Stem Cells and Cancer

**DOI:** 10.3390/genes8020045

**Published:** 2017-01-25

**Authors:** Alex Vassilev, Melvin L. DePamphilis

**Affiliations:** Eunice Kennedy Shriver National Institute of Child Health and Human Development, Bldg. 6A, Room 3A15, 6 Center Drive, Bethesda, MD 20892-2790, USA; vassilev@mail.nih.gov

**Keywords:** DNA re-replication, endoreplication, mitotic slippage, Geminin, teratoma, teratocarcinoma, embryonic stem cells, embryonal carcinoma cells, cancer stem cells, germ cell neoplasia

## Abstract

Cancers can be categorized into two groups: those whose frequency increases with age, and those resulting from errors during mammalian development. The first group is linked to DNA replication through the accumulation of genetic mutations that occur during proliferation of developmentally acquired stem cells that give rise to and maintain tissues and organs. These mutations, which result from DNA replication errors as well as environmental insults, fall into two categories; cancer driver mutations that initiate carcinogenesis and genome destabilizing mutations that promote aneuploidy through excess genome duplication and chromatid missegregation. Increased genome instability results in accelerated clonal evolution leading to the appearance of more aggressive clones with increased drug resistance. The second group of cancers, termed germ cell neoplasia, results from the mislocation of pluripotent stem cells during early development. During normal development, pluripotent stem cells that originate in early embryos give rise to all of the cell lineages in the embryo and adult, but when they mislocate to ectopic sites, they produce tumors. Remarkably, pluripotent stem cells, like many cancer cells, depend on the Geminin protein to prevent excess DNA replication from triggering DNA damage-dependent apoptosis. This link between the control of DNA replication during early development and germ cell neoplasia reveals Geminin as a potential chemotherapeutic target in the eradication of cancer progenitor cells.

## 1. Cancer

### 1.1. What Is Cancer?

Cancer refers to tumors and other forms of abnormal tissue growth (neoplasia). In general, cancers exhibit 10 hallmarks [1,2]: self-sufficiency in growth signals, insensitivity to anti-growth signals, evasion of apoptosis, unlimited proliferation, sustained angiogenesis, invasion of local tissues and metastasis to distant sites, utilization of abnormal metabolic pathways to generate energy (e.g., the Warburg hypothesis), evasion of the immune system, genome instability, and chronic inflammation. Thus, cancer cells are distinct from normal cells in their ability to proliferate under conditions where normal cells cannot, and to migrate and initiate growth in new locations. 

### 1.2. What Is the Likelihood of Developing a Cancer?

**Figure 1:**
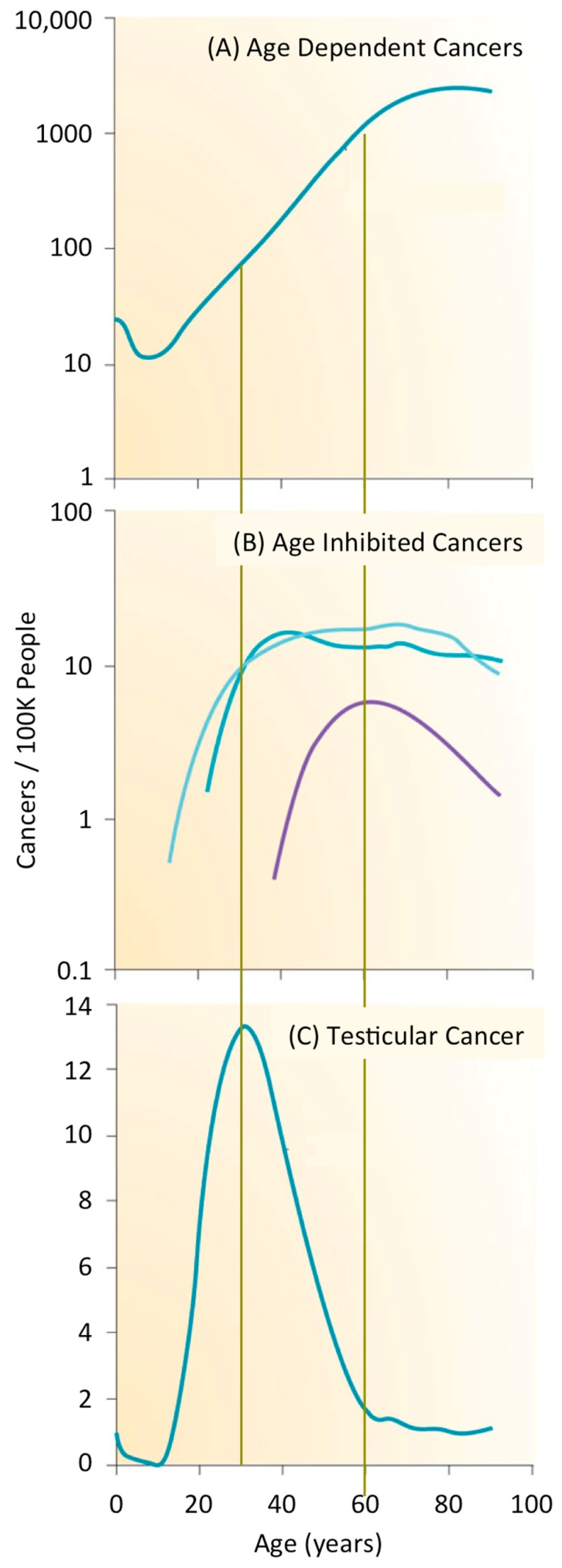
The incidence of various cancers is a function of age. The cancer frequency per 100,000 people as a function of age from the entire United States population during the years 1999 through 2009 [3]. Data are for males and females combined, except where indicated. (**A**) Age dependent cancers are represented by cancers of the stomach, colon, lung, breast (female), prostate, bladder, brain, lymphomas, leukemias, and melanoma; (**B**) Age inhibited cancers are represented by tonsil (purple), thyroid (light green), cervix and uterine (dark green); (**C**) Germ cell neoplasias are represented by testicular cancer.

Cancer is second only to heart disease as the leading cause of death in the USA and worldwide. Cancer accounts for 68% of all deaths from non-communicable diseases worldwide, and 23% of all deaths in the USA. In 2016, about 1/200 people will be diagnosed with cancer; about 35% will die from the disease (Centers for Disease Control). Cancers can be divided into three groups on the basis of age [3]. After sexual maturity, the incidence of cancer increases exponentially with age such that about 1% of men and women will develop a cancer by age 60 (Figure 1A). Thus, most cancers are a disease of aging for which the accumulation of genetic mutations and chromosome aberrations are primarily responsible, although other ageing-associated processes could also contribute. For example, accumulation of senescent cells and increased inflammation appear to promote cancer initiation and growth. In contrast, the frequency of a subset of cancers is inhibited with age (Figure 1B). Vascular ageing and a decline in growth hormone levels appear to reduce initiation and growth of cancers. The rate of thyroid, cervix, and uterine cancers is constant after about 30 years of age, and the frequency of tonsil cancer is primarily confined to people about 60 years of age. However, most striking are germ cell cancers that appear primarily among newborns, adolescents, and young adults. The frequency of testicular cancer, for example, peaks at about 35 years of age when it occurs in about 0.01% of men (Figure 1C).

### 1.3. What Are the Origins of Cancer?

There are two hypotheses about the origins of human cancer. The first is that cancer results from genetic mutations that are either inherited or acquired through errors in DNA replication and environmental insults [4]. This theory would account for the correlation between aging and the risk of developing a cancer [5]. The second theory is that cancer results from cancer stem cells (CSCs) that retain their ability to proliferate repeatedly without losing their ability to initiate uncontrolled growth, leading to cancer [6,7]. All cancer cells can proliferate under conditions where normal cells do not, but only CSCs can initiate a tumor de novo. Definitive evidence for the existence of CSCs was first reported for leukemia [8,9] and then extended to solid tumors that occur in the breast [10], brain [11,12], prostate [12], and colon [13]. These two theories are not mutually exclusive, because CSCs might arise during mammalian development through the accumulation of genetic mutations. Alternatively, CSCs might represent quiescent stem cells that eventually awaken within an alien environment (i.e., ectopic site) and therefore respond to proliferation and migration signals for which they are not developmentally programmed to respond.

### 1.4. Intrinsic versus Extrinsic Risk Factors

What is well established is that the frequency of cancer among different tissues and organs is distributed unevenly across the body both in time and space; some tissue types give rise to human cancers millions of times more often than other tissue types. What is not clear is the contribution of intrinsic risks for developing a particular cancer during one’s lifetime, such as random mutations that occur during stem cell proliferation, versus the contribution of extrinsic risks, such as viruses, chemical carcinogens, and radiation. 

**Figure 2:**
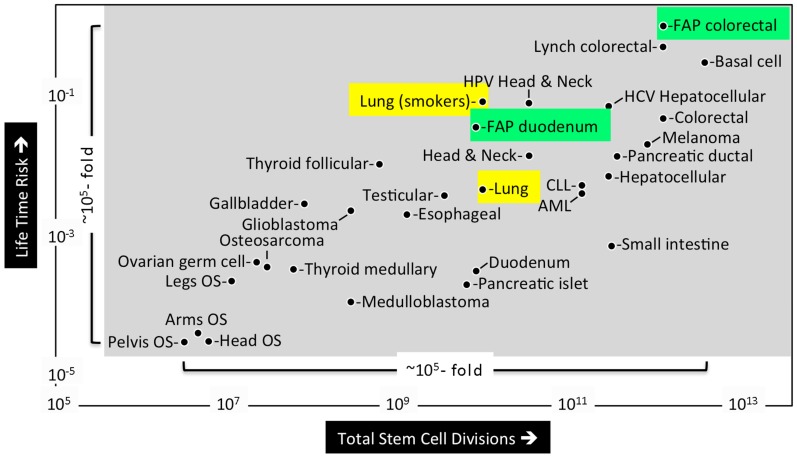
Differences in cancer risk among different tissues can be explained by the total number of stem cell divisions in those tissues [5]. The total number of cell divisions during the average lifetime of a human multiplied by the number of stem cells in a tissue (*x* axis) was plotted against the lifetime risk for cancer of that tissue type (*y* axis) for 31 tissue types in which stem cells had been quantitatively assessed. Only 9 out of 31 cancers were influenced significantly by extrinsic factors (example smoking (yellow)). Hereditary risk factors occurred more frequently in some tissues than in others (example, FAP gene mutations (green)). Abbreviations are Osteosarcoma (OS), Familial Adenomatous Polyposis (FAP), Hepatitis C virus (HCV), Human Papillomavirus (HPV), Chronic Lymphocytic Leukemia (CLL), and Acute Myeloid Leukemia (AML).

Mathematical analysis of published data led Tomasetti and Vogelstein to conclude that the lifetime risk of cancers is strongly correlated with the total number of divisions of the normal self-renewing cells maintaining that tissue’s homeostasis [5]. These tissue progenitor cells must arise from the tissue specific stem cells produced during embryonic development (discussed below). The lifetime risk for cancer was plotted against the number of stem cell divisions in 31 tissue types for which stem cells have been quantitatively assessed (Figure 2). The results revealed a dramatic correlation between these two parameters over five orders of magnitude. Moreover, they revealed that cancers with known hereditary risk factors occurred more frequently in some tissues than in others. For example, Familial Adenomatous Polyposis Coli gene mutations were ~30-fold more likely to cause colorectal cancer than duodenum cancer, apparently because the colon requires ~150-times as many stem cell divisions as does the duodenum. In contrast, extrinsic risk factors, such as smoking, Hepatitis C virus, or Human Papillomavirus significantly increased the risk of cancer in the lungs, liver, and head/neck, respectively. For example, people who smoke cigarettes are ~18-times more likely to develop lung cancer. These results suggest that only ~30% of the variation in cancer risk among tissues is attributable to environmental factors or inherited predispositions. The majority of cancers result from random mutations arising during DNA replication in the normal stem cells required during development and tissue maintenance.

Distinguishing the contributions of intrinsic from extrinsic risks is important not only for understanding the disease but also for designing strategies to limit the mortality it causes. Thus, it is not surprising that the Tomasetti and Vogelstein hypothesis ignited a firestorm of controversy. Six letters to the editor of Science stated that they had understated the role of environmental factors, that many types of tumors were not considered, that the role of chance was overstated, that current evidence shows some cancers are preventable, that most cancers are caused by multiple overlapping factors, and that the selection criteria for which cancers were selected for this study were not sufficiently robust (discussed in [14]). In the year that followed, at least 20 opinion pieces were published in many different journals, both favorable and critical. Remarkably, using the same data analyzed by Tomasetti and Vogelstein, Wu and co-workers concluded that the correlation between stem-cell division and cancer risk does not distinguish between intrinsic and extrinsic factors [4]. They concluded that endogenous mutation rates by intrinsic processes could not account for the observed cancer risks, and that 70% to 90% of the common cancers are caused by extrinsic factors.

**Figure 3:**
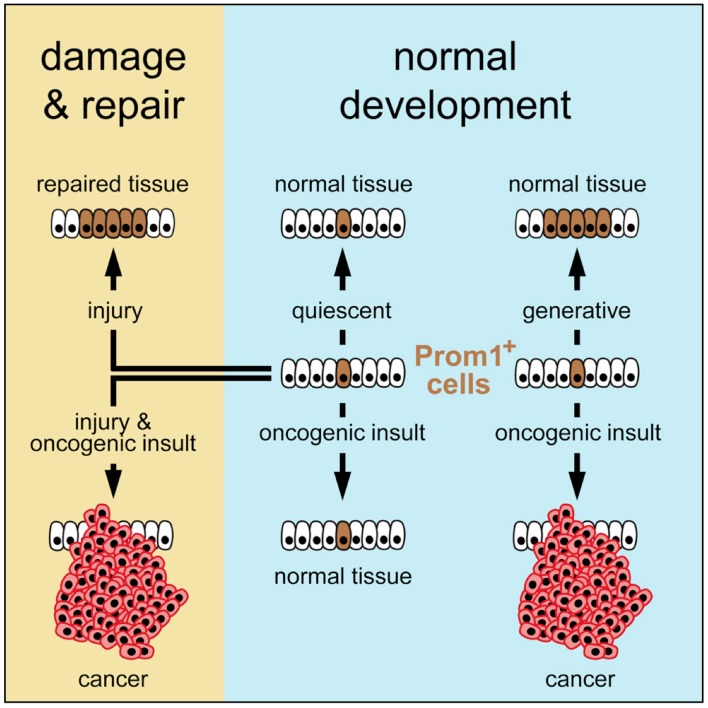
The generative capacity of an organ’s stem cells determines the life-long risk for developing cancer in that organ [15]. In addition, extrinsic factors converge specifically on stem cells to induce mutations and/or tissue damage that provokes proliferative repair. Tissue specific susceptibility of stem cells to induced mutations and their intrinsic, or damage-induced proliferative capacity, create a ‘‘perfect storm’’ that ultimately determines organ cancer risk.

To resolve this conundrum, Zhu and co-workers mapped the frequency of cancer in various organs of mouse neonates and adults [15]. Their strategy was to circumvent the need to consider extrinsic factors by mapping the fate of stem cells that already contained oncogenic risk factors, thereby revealing only the role of cancer driver mutations together with the number of stem cell divisions that occurred in each organ over time. They engineered mice to express a tamoxifen–dependent *ErCre* recombinase and *LacZ* reporter driven by the promoter of an endogenous cell surface antigen (Prom1) that is common to stem cells and distributed widely among tissues and organs. These ‘Prom1+ mice’ were mated with mice harboring ErCre-dependent conditional knockout alleles that activate a lineage tracer together with a series of oncogene and tumor suppressor alleles in cells that express the Prom1 gene. Their results revealed that the risk of an organ developing cancer is significantly associated with the life-long generative capacity of its mutated cells (Figure 3). If a stem cell was quiescent, it did not produce a cancer, regardless of the presence or absence of oncogenic mutations. If stem cells underwent multiple generations, then the frequency of cancer was greatly dependent on the number of stem cell divisions as well as the presence of an oncogenic driver mutation. This relationship was true in the presence of multiple genotypes and regardless of the developmental stage, strongly supporting the notion that the frequency of stem cell proliferation dictates cancer risk among organs, as suggested by Tomasetti and Vogelstein.

Nevertheless, extrinsic factors such as tissue damage could play a leading role. Oncogenic mutations that had been introduced into the stem cells of normal adult livers were insufficient to induce tumors, because these cells were quiescent. However, when partial hepatectomy induced cell proliferation, the transformed stem cells produced a cancer. Thus, the carcinogenic properties of some extrinsic factors might relate solely to their induction of local tissue damage and activation of cell repair, thereby accelerating cell proliferation, which promotes cell transformation. In this model, organ cancer risk is determined by a combination of factors: the intrinsic proliferative capacity of the stem cell population, the incidence of local tissue damage that induces cell proliferation, and the susceptibility of these cells to mutations that can transform them into cancer.

### 1.5. Clonal-Evolution of Cancer

With rare exceptions, spontaneous tumors originate from a single cell. Nevertheless, at the time of clinical diagnosis, the majority of human tumors display startling heterogeneity such as expression of cell surface receptors, proliferation, and angiogenesis, for which there is strong evidence for the co-existence of genetically divergent tumor cell clones within tumors [16]. Such tumor heterogeneity can be identified by differences in cell morphology, genomic DNA, and gene expression profiles that allow tumors to be classified into subtypes. In the ‘clonal-evolution model’ [17], the types of mutations will vary as a cancer develops, so that individual cancer cells become more transformed and aggressive. In fact, sequencing DNA from cancer patients has confirmed the subsequent and independent accumulation of genetic mutations during metastasis of the original tumor [18,19]. Phylogenetic analysis of the mutations carried by individual metastatic sites suggest branched tumor evolution with 63% to 69% of all somatic mutations not detectable across every tumor region [18].

### 1.6. Take-Home Lesson

Cancer is an endemic disease that results from an accumulation of genetic defects in the form of nucleotide mutations, chromosomal rearrangements, polyploidy, and aneuploidy. Whether the bulk of these genetic defects are created intrinsically through errors in DNA replication during cell proliferation, or extrinsically by radiation, carcinogenic chemicals or viruses remains a matter of intense investigation. That said, DNA replication and stem cells are clearly major contributors.

## 2. DNA Replication and Cancer

**Figure 4:**
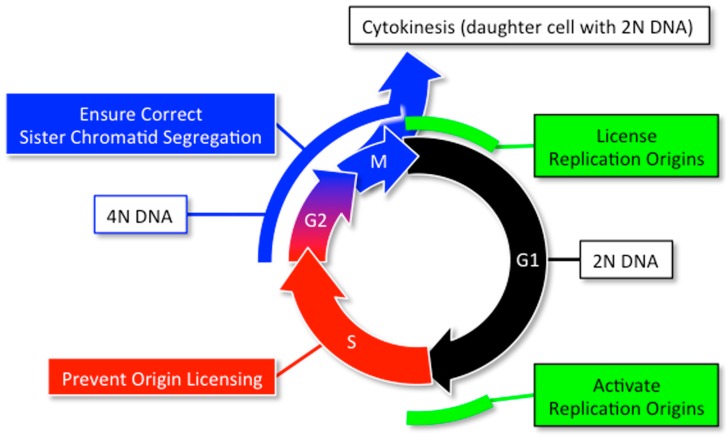
The mammalian mitotic cell division cycle consists of five phases. During G1-phase the cell grows in size and licenses its replication origins by assembling prereplication complexes in preparation for nuclear DNA replication (termed ‘origin licensing’). S-phase begins when the licensed replication origins are organized further into preinitiation complexes that are activated by two separate protein kinase activities to begin bidirectional DNA replication. G2-phase is a brief period of time between the end of nuclear DNA replication and the beginning of mitosis (termed M-phase). Mitosis is the separation of the homologous pairs of chromosomes into two identical nuclei, each with 2N DNA content. Cytokinesis is the separation of the binucleate cell into two cells. To insure that the daughter cells each receive one and only one copy of the genome, origin licensing is confined to the transition from M to G1 phase, and origin activation is confined to S-phase.

The prime directive that drives the mitotic cell division cycle is that the nuclear genome is duplicated once, but only once, each time a cell divides [20]. Robust regulatory networks normally restrict nuclear DNA replication to one complete duplication of the genome each time a cell divides (Figure 4). The assembly and activation of replication proteins at selected sites along nuclear DNA is restricted to the M to G1 phase transition and the G1 to S phase transition, respectively. Origin licensing is actively prevented during the S to early M-phase period, and mechanisms are in place during G2-phase through cytokinesis to ensure that each daughter cell receives one nucleus with two complete sets of chromosomes. 

Exceptions to this rule are rare, and those that do occur are developmentally regulated to produce terminally differentiated, viable, nonproliferating cells by meiosis, failed cytokinesis, endomitosis, or endoreplication (Box 1).

**BOX 1. Developmentally Regulated Changes in Ploidy**

**Meiosis** - DNA replication is followed by two rounds of mitotic cell division in the absence of nuclear DNA replication to produce four cells, each with half the number of chromosomes as the original parent cell. Haploid germ cells (sperm and oocytes) arise by diploid germ cells undergoing meiosis.

**Failed Cytokinesis**—Myocardiocytes and hepatocytes result from a failed cytokinesis that produces a binucleate tetraploid cell (one cell with two nuclei, each with 2N DNA content) [21,22]. Binucleate tetraploid cells can complete a successful cell cycle plus mitosis, generating mononucleate tetraploid cells where each nucleus is 4N. Reiteration of these events accounts for the rare octoploid and hexadecaploid cells.

**Endomitosis**—Megakaryocytes are bone marrow cells responsible for the production of blood thrombocytes (platelets), which are necessary for blood clotting. Thrombopoietin promotes the growth and development of megakaryocytes from their hematopoietic stem cell precursors (megakaryoblasts) by triggering endomitosis, repeated cycles of DNA replication followed by entrance into mitosis without cytokinesis. This results in a single polylobulated nucleus containing multiples of 4N DNA (e.g., 8N, 16N, 32N, etc.) that eventually undergoes platelet formation. Endomitosis occurs because of a defect in late cytokinesis that results in incomplete formation of the cleavage furrow, a contractile ring consisting of myosin II and F-actin that generates the mechanical forces necessary for cell separation [23,24]. Down-regulation of the *ECT2*, a gene that is essential for cytokinesis, is required for polyploidization beyond 4N [25]. In addition, up-regulation of G1-phase components, such as cyclin E, might be important in promoting multiple cycles of endomitosis [26].

**Figure 5:**
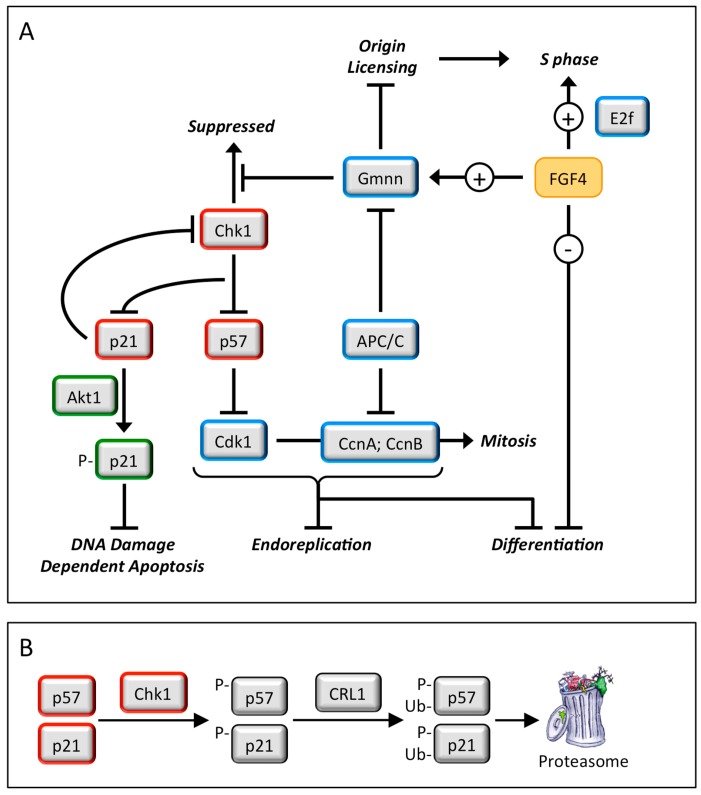
The fibroblast growth factor-4 (FGF4) signal transduction pathway governs trophoblast proliferation and differentiation. FGF4 (and probably other mitogenic proteins as well) is essential for trophoblast proliferation. This mitogenic activity is likely mediated by E2f-dependent gene expression [28,29], and possibly directed at regulating the activity of the anaphase-promoting complex (APC) [30]. FGF4 deprivation results in down-regulation of Geminin activity to a level that maintains endocycles [31], but that does not prevent down-regulation of Chk1 protein. The loss of Chk1 kinase activity results in the expression of two CDK-specific inhibitors, p57 and p21 [32]. The p57 protein prevents the onset of mitosis by selectively inhibiting Cdk1 activity, thereby triggering the first round of endoreplication [33,34]. This event activates the G1-phase APC•Cdh1 ubiquitin ligase, which targets Geminin, Cyclin B, and Cyclin A proteins for degradation, thereby allowing licensing of replication origins and the onset of S-phase without passing through mitosis [22,35]. Inhibition of Cdk1 triggers both endoreplication and trophoblast stem cell (TSC) differentiation. In the absence of p57, FGF4 deprivation produces multinucleated trophoblast giant cells (TGCs), revealing the existence of alternative mechanisms to trigger TSC differentiation [34]. Endocycles also require p57, which is expressed during G-phase and then suppressed during S-phase to allow sequential assembly and activation of pre-replication complexes [34]. Geminin maintains endocycles by preventing DNA re-replication. The p21/Cdkn1a protein localizes to the cytoplasm in TGCs where it prevents DNA damage induced apoptosis [36]. It might also maintain suppression of Chk1 by reducing Chk1 RNA levels [37], as observed during FGF4 deprivation [32].

**Figure 6:**
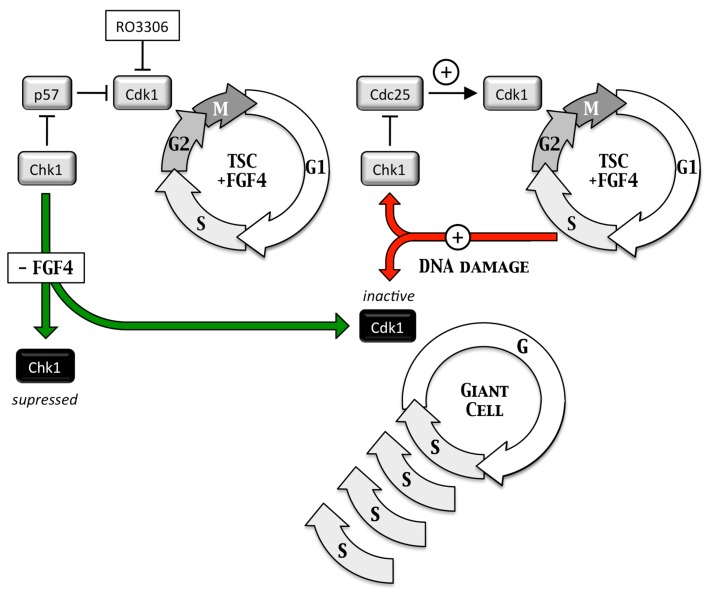
Inhibition of Cdk1 activity triggers endoreplication in trophoblast cells. Selective inhibition of Cdk1 activity in trophoblast stem cells by RO3306, FGF4-deprivation, or induction of DNA damage triggers multiple S-phases without an intervening mitosis or cytokinesis to produce giant cells with a single enlarged nucleus containing as many as several hundred copies of each chromosome [34].

**Endoreplication**—Trophoblast giant cells are essential for implantation of the embryo into the uterine endothelium and subsequent placentation. They arise when trophoblast stem cells are depleted of fibroblast growth factor 4 (FGF4), which triggers depletion of CHK1 protein kinase, which allows p57 to inhibit CDK1•CcnB, the enzyme essential to initiate and maintain mitosis, and p21 to inhibit DNA damage dependent apoptosis (Figure 5) This causes trophoblast stem cells to under undergo multiple S-phases in the absence of an intervening mitosis (termed endoreplication or endocycles) without proliferating and without dying [27]. The result is nonproliferating trophoblast giant cells each with a single nucleus containing integral multiples of 4N DNA content (e.g., 8N, 16N and 32N). Because this pathway is dependent on the DNA damage response gene, CHK1, DNA damage in trophoblast stem cells can also produce trophoblast giant cells (Figure 6).

Nevertheless, the seeds to cancer are planted by the errors that occur during DNA replication. When these seeds are planted within genes that regulate genome duplication, they can initiate a cancer by creating an oncogene or inactivating a tumor suppressor gene. When these seeds trigger aberrant forms of DNA replication in the form of unscheduled endoreplication and DNA re-replication (Box 2), they result in polyploidy or aneuploidy, which drives the ‘mutator phenotype’ in cancer cells that leads to more aggressive, drug-resistant, forms of cancer. In fact, genes that prevent missegregation of sister chromatids during mitosis also prevent unscheduled endoreplication. Therefore, fluctuations in the levels of these genes would promote the frequency of missegregation through excess genome duplication.

**BOX 2. Aberrant Forms Of Genome Duplication**

**Mitotic Slippage**—Drugs that inhibit microtubule dynamics arrest proliferation when cells enter mitosis [38]. However, cells do not remain in mitosis indefinitely, because the anaphase-promoting complex (APC) is activated within several hours [39,40]. Activation of the APC allows cells to re-enter G1-phase as tetraploid cells with either a single enlarged nucleus or several micronuclei [41]. This aberrant event is termed mitotic slippage, and it generally results in DNA damage and apoptosis. Tetraploid cells can also be induced by metabolic stress, wound healing, ageing, and senescence [42].

**Unscheduled Endoreplication**—Unscheduled endoreplication occurs in two ways. First, drug induced mitotic slippage produces tetraploid cells, which might or might not enter S-phase. However, cells lacking a G1 checkpoint, such as Tp53 or Rb1 deficient cancer cells, more easily proceed into S-phase, thereby producing a single cell with a giant nucleus containing 8N DNA [43,44,45]. Alternatively, suppressing expression of genes such as CDK1•CCNB [34,46,47,48] that are essential for entrance into mitosis, or for genes that are essential for cytokinesis [49] results in repeated rounds of nuclear DNA replication that produce cells with a single nucleus containing an integral multiple of 4N DNA content (e.g. 8N, 16N or 32N).

**DNA Re-replication**—Once S-phase begins; origin licensing must be prevented until mitosis is completed. Otherwise, regions of DNA that have already replicated during S-phase will be replicated a second time during the same S-phase. This aberrant form of DNA replication, termed DNA re-replication, results in partial replication of regions of nuclear DNA. These cells contain a highly variable and heterogeneous nuclear DNA content ranging from 4N through 8N or even greater. DNA re-replication produces additional replication forks that are easily converted into double-strand DNA breaks, which are difficult to repair and therefore trigger apoptosis. Those cancer cells that suppress apoptosis will become aneuploid.

**Figure 7:**
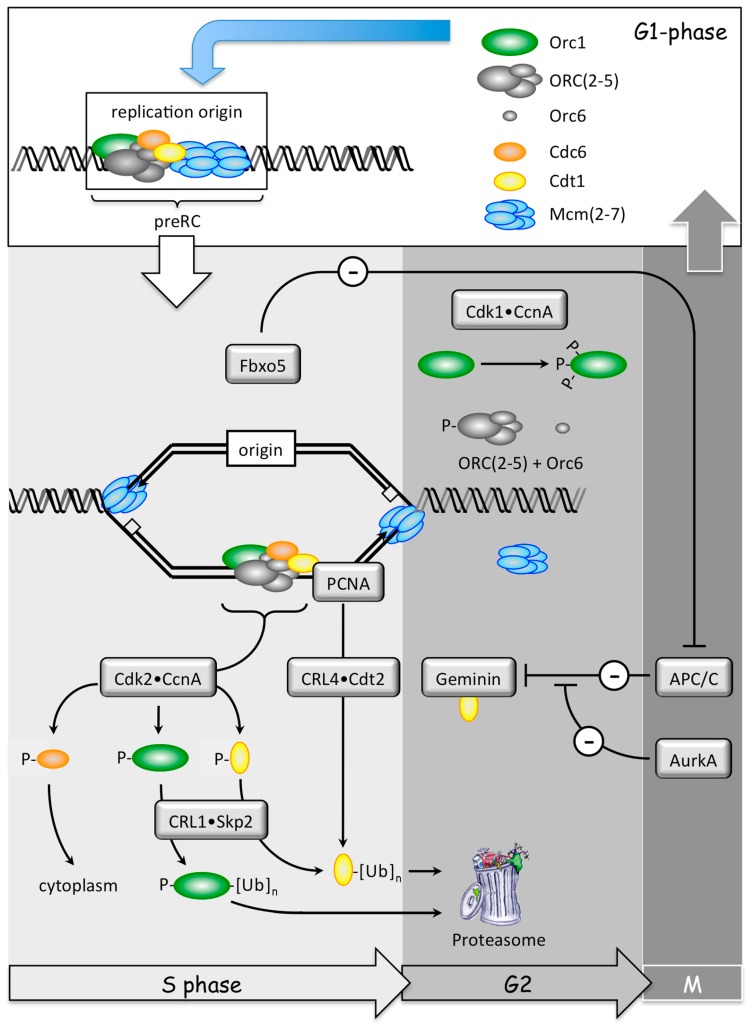
The origin recognition complex (ORC) and the CDT1 cycles prevent DNA re-replication. The ‘ORC cycle’ begins when the Orc1 subunit of the origin recognition complex [ORC(1–6)] is selectively targeted during S-phase for inactivation by post-translational CDK-dependent phosphorylation by Cdk2•CcnA and then ubiquitin-dependent degradation by CRL1•Skp2 [51,92]. The first step inhibits ORC activity; the second destroys it. Since the Orc1 subunit is essential for ORC binding to DNA, loss of the Orc1 subunit results in destabilization of the remaining ORC subunits [93,94]. Since Cdc6 protein binding to DNA is dependent on ORC(1–6), destabilization of the ORC-DNA interaction will destabilize the Cdc6-DNA interaction. Cdc6 then becomes a target for phosphorylation by Cdk2•CcnA, which results in its nuclear exclusion. These events should prevent premature licensing of replication origins during S-phase. Reassembly of prereplication complexes (preRCs) appears to be triggered by the Orc1 subunit during the anaphase to G1-phase transition [95]. Orc1, the ORC(2–5) core complex, Orc6, and Cdc6 associate with DNA to form a ‘helicase loader’ [53,96,97]. Cdt1 protein then allows loading of the heterohexamer protein complex Mcm(2–7), the mammalian DNA helicase, to complete ‘origin licensing’.

At least seven concerted pathways exist that prevent DNA re-replication in mammalian cells by inactivating the helicase loader, thereby preventing both the reloading of MCM helicases at activated replication origins and the licensing of new replication origins (Figure 7). These pathways, which can be categorized as the ‘ORC cycle’ and the ‘Cdt1 cycle’, exist in flies, frogs, nematodes, and mammals [50,51,52,53]. DNA re-replication can be readily induced in cells derived from human cancers either by depletion of Geminin, or by depletion or inhibition of Cullin-based ubiquitin ligases [54,55].

The essential distinction between developmentally regulated changes in ploidy (Box 1) and aberrant forms of genome duplication (Box 2) is genome stability; developmentally regulated changes in ploidy result in stable haploid or polyploid cells that do not proliferate, whereas aberrant forms of genome duplication result in inherently unstable polyploid cells precisely because they do proliferate. For example, experimentally induced tetraploid cells arrest their cell cycle in G1 phase without completing mitosis and cytokinesis, but in the absence of a TP53 or Retinoblastoma dependent checkpoint, tetraploid cells continue into S phase, which results in cell death or aneuploidy [43,45,56,57,58,59,60,61].

### 2.1. Normal DNA Replication Produces Genetic Mutations

The replicative DNA polymerases are remarkably precise in ensuring correct pairing of nucleotides during DNA synthesis; nevertheless, they make mistakes at a rate of about 1 per every 100,000 nucleotides [62]. Given that the human diploid genome is 6.16 billion (6.16 × 10^9^) bp in size, the replicative DNA polymerases introduce 60,000 errors every time the cell divides. However, DNA polymerase proofreading and mismatch nucleotide repair enzymes correct the vast majority of these mistakes, thereby reducing the observed mutation rate in humans to one error for each 10^9^ to 10^10^ nucleotides polymerized, or about 0.3–3 mutations per genome per duplication [63,64]. Since converting a fertilized egg into an adult of some 100 trillion cells requires about 47 genome duplications, the simple act of human development results in from 14 to 140 genomic mutations. In addition, at least another 10–100 mutations per genome arrive at conception as a result of the accumulation of mutations between fertilization of the egg until formation of the next generation of gametes [65]. 

The major source of mutations that trigger human disease results from the simple fact that most of the cells in our body are replaced from every few days to every few weeks, which results in trillions upon trillions of additional cell divisions during a human lifetime [65]. For example, in those tissues where cells are replaced every other day for 71 years (average human life span), stem cells will have replicated their nuclear DNA approximately 12,500 times, thereby introducing 7500 mutations, such mutation rates account for the high frequency of mutations observed in human cancers. External environmental factors can further increase the error rate during DNA replication by causing DNA damage or by stimulating cell proliferation to repair damaged tissue.

### 2.2. Cancer Cells Have Exceptionally High Levels of Genetic Alterations

The frequency of chromosomal abnormalities and nucleotide sequence alterations in the nuclear DNA of human cancers far exceeds those in normal human cells [64]. For example, analysis of 58 colorectal tumors revealed that at least 11,000 individual genomic events had occurred in each tumor cell, suggesting that the onset of genome instability is an early event in tumor progression that acts as a facilitator and not a consequence of malignancy [66]. Moreover, pre-cancerous colonic polyps contained similar frequencies of genomic alterations, indicating that they are the initiator, not the consequence, of malignancy. 

Remarkably, only a few of these mutations, termed ‘cancer driver mutations’, occur in oncogenes and tumor suppressor genes, thereby conferring selective growth advantages to the cancer cell in which they occur. The remaining thousands of mutations are ‘passengers’ that occurred coincidentally during the large number of cell divisions associated with the neoplastic process [67,68]. Estimates for the number of mutations required for a normal human cell to progress to an advanced cancer, based on the relationship between age and incidence, suggest that six or seven driver mutations are required. More recently, an estimate based on the incidence within different groups of patients with the same cancer type compared with their somatic mutation rates concluded that only three sequential mutations are required to develop lung or colon adenocarcinomas [69]. But this simple view cannot account for the broad phenotypic and functional heterogeneity that are hallmarks of cancers.

### 2.3. Polyploidy Promotes Aneuploidy Which Promotes Cancer

The increased potential of neoplastic cells to evolve more aggressive sub-clones is linked directly to the extent of their genome instability [18,19], and genome instability is linked directly to aneuploidy, and aneuploidy is promoted by polyploidy [70]. The reported accelerated ability of preneoplastic and neoplastic cells to generate new genetically diverse phenotypic variants or genomic instability has been long observed as an integral part of cancer development. Cell lines derived from cancers usually demonstrate high rates of genetic instability with widely varying chromosomal content that changes continuously with each mitotic cell division, resulting in a cellular heterogeneity that is restored quickly after clonal selection [71]. In the vast majority of cancers, genome instability is manifested as polyploidy or aneuploidy [72]. Polyploid cells contain multiple copies of the complete genome, whereas aneuploid cells contain either more or fewer copies than normal cells of either chromosomal regions or complete chromosomes.

Aneuploidy can result from defects in cell cycle events, such as DNA replication, attachment of microtubules to chromosomes, spindle assembly checkpoint, sister chromatid cohesion, centrosome duplication, and telomere maintenance [73,74]. The current consensus is that aneuploidy develops progressively from the diploid state through the accumulation of mutations that result in genome instability; chromosome gain and loss result from missegregation during mitosis [75] and that tetraploidy promotes aneuploidy [70,76]. The frequency at which aneuploidy occurs is accelerated by passing through polyploidy. Aneuploid cells have been identified in up to 80% of human cancers, particularly in solid tumors, where they are associated with a poor prognosis for recovery [70,77,78,79]. Sequencing nuclear DNA from tumors revealed that at least one in three tumors had transitioned through a polyploid state during its development, providing strong support for the hypothesis that tumorigenesis is accelerated by transitioning through the inherently unstable polyploid state [80]. In fact, tetraploid cells induced in vitro from cell lines derived from either cancer or non-transformed cells transitioned to aneuploidy, chromosome instability, and increased resistance to chemotherapeutic drugs with higher frequency than their diploid counterparts [70]. Tetraploid cells stimulate tumorigenesis in mice, particularly if they lack Tp53 activity [81,82]. Therefore, polyploidy promotes aneuploidy and tumor formation.

The question often arises as to whether aneuploidy is a cause or a consequence of carcinogenesis. The weight of evidence supports the conclusion that aneuploidy enhances genetic recombination and defective DNA damage repair, thereby providing a mechanistic link between aneuploidy and genomic instability [83,84]. In effect, aneuploidy drives the ‘mutator phenotype’ associated with cancer [85]. The ‘mutator phenotype’ hypothesis accounts for the fact that mutations are much more common in cancer cells than in normal cells, and even increase with tumor expansion, by mutations that arise in a cancer cell that greatly accelerates carcinogenesis [64]. For example, mutations in DNA polymerases that increase the mutation rate, as well as mutations in DNA damage repair pathways that suppress their ability to correct mistakes during DNA replication would contribute to the overall mutation rate during cell division. 

### 2.4. Preventing Excess Genome Duplication Prevents Aneuploidy and Tumorigenesis

Excess genome duplication (EGD) arises when cells depend on fewer genes to prevent aberrant cell cycle events such as mitotic slippage, unscheduled endoreplication, and DNA re-replication. For example, some cancer cells rely solely on Geminin protein to prevent DNA re-replication [86,87] and the Fbxo5 protein to prevent degradation of Geminin during DNA replication. This would account for the fact that Geminin is over-expressed in many tumors, and the prognosis for recovery is inversely related to the level of Geminin expression [88,89]. Moreover, suppressing Geminin expression can prevent tumor growth [90,91]. Cells in which Geminin depletion does not induce DNA re-replication either rely upon alternative pathways (Figure 7) to prevent DNA re-replication [86], or else the level of depletion was insufficient. For example, the Geminin gene (*Gmnn*) is haplo-sufficient in cells for which *Gmnn* ablation reveals that it is essential for proliferation and viability [91].

The ‘CDT1 cycle’ begins when Cdt1 protein is targeted for ubiquitin-dependent degradation during S-phase by two independent pathways: CDK-dependent phosphorylation followed by ubiquitination of Cdt1-P by CRL1•Skp2, and PCNA-DNA-dependent ubiquitination of Cdt1 by CRL4•Cdt2. PCNA is the eukaryotic sliding clamp protein that facilitates DNA synthesis by DNA polymerases-δ and -ε. The ubiquitinated proteins are then degraded by the 26S proteasome. Cdt1 activity is also inhibited by binding to Geminin protein, but the importance of Geminin is confined primarily to late S, G2, and early mitosis [90,98]. These activities are available from S through early M-phase. As cells exit mitosis, Geminin and Cyclin A are ubiquitinated by the anaphase-promoting complex (APC/C), an activity that is inhibited specifically by Fbxo5/Emi1 during S to early M-phase. Geminin binding to Cdt1 and CDK-dependent phosphorylation of Cdc6 prevent Cdt1 and Cdc6 degradation by the APC/C during mitosis, thereby allowing them to participate in origin licensing as cells exit mitosis [99,100]. The Cdt1 cycle is critical at the beginning of animal development. 

**Figure 8:**
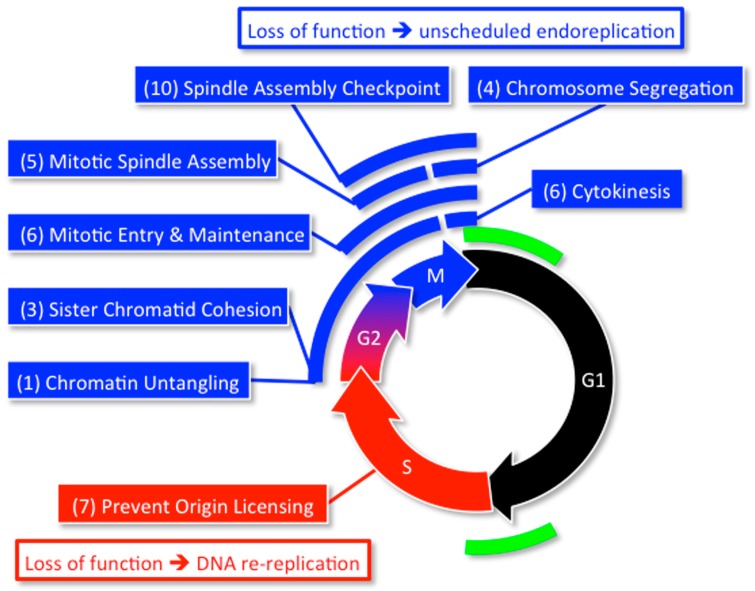
Specific cell cycle events associated with either DNA re-replication or unscheduled endoreplication. Forty-two genes (Table 1) participate in one or more of eight cell cycle events that restrict genome duplication to once per cell division [55]. Fluorescence activated cell sorting (FACS) analyses of HCT116 cells (±ZVAD, a specific inhibitor of apoptosis) transfected with small interfering RNAs (siRNAs) against the genes in Table 1 revealed that some cell cycle events (indicated in blue) prevented primarily unscheduled endoreplication whereas others prevented primarily DNA re-replication (indicated in red). Origin licensing refers to the assembly of prereplication complexes during the anaphase to G1-phase transition. Origin activation refers to the assembly of initiation complexes during the G1 to S-phase transition. Number of genes essential for each cell cycle event is in parenthesis.

Since high throughput screens for genes that affect the mitotic cell division cycle in HeLa cells and U2OS cells did not detect Geminin [101,102], it was likely that they missed other genes associated with excess genome duplication, as well. Therefore, a high throughput screen of about 95% of the human genome (21,584 genes) was carried out on the HCT116 colorectal carcinoma cell line [55]. This cell line is not only acutely sensitive to Geminin depletion [86,87], but it has a stable, near diploid, karyotype [103]. This screen revealed 42 genes (Table 1) that prevent EGD by participating in one or more of eight specific cell cycle events (Figure 8). These genes not only include those previously shown to restrict genome duplication to once per cell division, but 17 genes that were not identified previously in this capacity. 

Mouse models expressing either haplo-insufficient or hypomorphic alleles of various genes reveal that efficient expression of at least 14 of the 42 genes in Table 1 are essential to prevent chromosome instability and aneuploidy in vivo (Table 2). All 14 genes are involved in attaching sister chromatids to the mitotic spindle that is essential for segregating the sister chromatids into separate cells during cytokinesis.

PLK1 (Polo-like kinase 1) phosphorylates FBXO5 just before nuclear envelope breakdown, thereby targeting it for ubiquitin-dependent degradation [122]. This allows CDC20 to either activate the APC or to be sequestered by the ‘spindle assembly checkpoint’ (SAC), a mechanism that prevents the metaphase-anaphase transition until all chromosomes are successfully attach to the bipolar spindle with proper tension [123,124]. SAC consists of ‘sensor’ proteins such as Mad1, Bub1, and Mps1, a ‘signal transducer’ consisting of the ‘mitotic checkpoint complex’, and an ‘effector’ known as the anaphase promoting complex/cyclosome (APC/C). Prior to the metaphase-anaphase transition, SAC inhibits the ability of Cdc20 to activate the APC/C, which stabilizes Securin (a specific inhibitor of Separase, the protease responsible for triggering anaphase) and cyclin B (an essential component of active Cyclin B•CDK1, the enzyme responsible for initiating and maintaining mitosis). These two proteins delay the metaphase-anaphase transition. Once the correct metaphase spindle•chromosome attachments have been established, the spindle assembly checkpoint is inactivated and APC/C(Cdc20) ubiquitinates Securin and cyclin B, thereby targeting them for degradation. Separase removes the cohesin complex that binds sister chromatids together, and the cell undergoes anaphase.

TPX2, KIF11/Eg5/Kinesin-11 and AURKA/Aurora Kinase A are proteins required to assemble the mitotic spindle. TPX2 is a microtubule associated protein that is essential for spindle assembly and chromosome segregation during prometaphase [105]. TPX2 regulates the activity of KIF11, a kinesin that functions early in mitosis to push the spindle poles apart by pulling microtubules past one another. Suppression of KIF11 activity activates SAC, resulting in mitotic arrest [125]. TPX2 also stabilizes the active conformation of AURKA, which is required for building a bipolar spindle regulating centrosome separation and microtubule dynamics.

INCENP/Inner Centromere Protein, BIRC5/Survivin, CDCA8/Borealin, and AURKB/Aurora kinase B are the four proteins that comprise the ‘chromosome passenger complex’. In the absence of complete kinetochore-microtubule attachments, the chromosome passenger complex promotes the recruitment of the ‘mitotic checkpoint complex’, consisting of the proteins MAD2L1, BUB1B, BUB3, and CDC20, to the kinetochore in a series of events catalyzed by the TTK/Mps1 protein kinase [126]. Depletion of any one of the chromosome passenger complex subunits, or BUB3, BUB1B, MAD2L1, or TTK proteins, results in excess genome duplication in vitro, and aneuploidy in vivo (Table 1 and Table 2). Aneuploidy is generally accompanied by increased tumorigenesis. Centromeric cohesin is preserved until metaphase by protein phosphatase 2A, which is targeted to centromeres by SGOL1/Shugoshin-like [127]. ESPL1 is the protease responsible for triggering anaphase by removing the cohesin complex that binds the sister chromatids together. 

### 2.5. Excess Genome Duplication (EGD) Promotes Aneuploidy

The fact that genes identified in vitro as essential for prevention of EGD are also essential for prevention of aneuploidy and tumorigenesis in vivo reveals that haplo-insufficient genes that are essential to prevent both missegregation of sister chromatids and EGD drive cells towards aneuploidy by forcing them to become polyploid as well. Clearly, missegregation alone can produce aneuploid cells, because disrupting SAC by depleting BUB3, BUBR1, OR MAD2 in mouse oocytes increases the incidence of aneuploidy under conditions in which nuclear DNA replication does not occur [115,116,128,129]. However, at least one third of cancers pass through a polyploid stage [80], and formation of tetraploid cells increases the frequency of aneuploidy [70,76].

Once a mutation in an essential EGD prevention gene allows aneuploidy and genome instability, the associated accelerated mutation rate will allow faster accumulation of cancer driver mutations and accelerate tumorigenesis. Revealing the importance of such aneuploidy prevention genes in cancer development is hindered by the fact that multiple proteins might work together to maintain an event essential for prevention of EGD. For example, each of the four subunits of the chromosome passenger complex is required to restrict genome duplication to once per cell division in a colon cancer cell line [55], and each of them is essential to prevent aneuploidy and polyploidy during mouse development [108,109,110,111]. Moreover, the experiments with haplo-insufficient or hypomorphic alleles of mitotic checkpoint components in mouse models reveal that EGD prevention genes need not be inactivated completely to induce chromosome instability and aneuploidy. Thus, identification of the importance of such events in tumorigenesis is technically challenging given the expected wide multitude of function related gene coding or expression modulating promoter mutations in multiple genes that can lead to inactivation of a single EGD prevention event. Since different cancers carry different sets of mutations, cells isolated from different cancers might well rely upon different sets of genes to prevent EGD during cell proliferation. Whether or not genes exist that prevent chromosomal loss without preventing EGD remains to be determined.

The importance of a particular gene in preventing EGD also depends on checkpoint control mechanisms. In the absence of TP53 function, the extent of EGD increases. The TP53 tumor suppressor pathway, which is activated in response to cellular stress or DNA damage, participates in multiple pathways that regulate cell cycle progression, promote apoptotic death, and prevent tetraploid cells from entering S-phase [130]. Remarkably, the TP53 pathway is not functional in most human cancers [131]. In some cells, the function of TP53 is inactivated directly by mutations in the TP53 gene, whereas in other cells the function of TP53 is inactivated indirectly by changes in the cellular proteins that interact either with TP53, or by TP53 binding to viral proteins [132]. Studies using isogenic cancer cells differing only by the presence of a functional TP53 gene have revealed that a functional TP53 mediated DNA-damage response reduces significantly the extent of EGD [55]. Inhibition of apoptosis with a pan-caspase inhibitor mimicked the effect of TP53 elimination, thereby confirming that EGD causes DNA damage-induced apoptosis mediated by the TP53 pathway. 

### 2.6. Take-Home Lesson

DNA replication over trillions of cell divisions clearly can provide sufficient genetic mutations to trigger the cancer driver mutations and initiate carcinogenesis. Furthermore, fluctuations in the levels of a small number of critical genes can result in excess genome duplication. Those genes that are involved in segregation of sister chromatids during mitosis not only prevent aneuploidy by preventing missegregation, but they also prevent excess genome duplication, which promotes aneuploidy and thereby amplifies the problem. Thus, a ‘mutator phenotype’ arises that allows the tumor’s environment to select more aggressive forms of cancer. On the other hand, unscheduled DNA replication events generally result in DNA damage, a DNA damage response and if the damage cannot be corrected, then apoptosis occurs. Therefore, one or more of the genes that prevent excess genome duplication might also represent the ‘Achilles’ heel’ of specific cancers. As we shall see, geminin is such a gene.

## 3. Stem Cells and Cancer

The sequence of events during mammalian development and the rise of stem cells has been elucidated most extensively in mice [133]. Stem cells are recognized as cells that can proliferate repeatedly while retaining their ability to differentiate into specific cell types (termed ‘self-renewal’; [134]). They are commonly referred to according to the number of different cell lineages to which they give rise. Thus, unipotent cells give rise to a single cell lineage and multipotent cells give rise to multiple cell lineages, but only those cells that can give rise to all of the cell lineages in the embryo and adult are termed pluripotent, and only those that give rise to the placenta as well as the embryo are termed totipotent [135]. The unipotent and multipotent 'tissue specific stem cells' can give rise to cancer through mutations that occur during the generations of DNA replication required to produce and maintain a particular tissue or organ. The ‘pluripotent stem cells’ that begin mammalian development could produce cancers directly by simply ending up in the wrong place at the wrong time during mammalian development. 

### 3.1. Tissue Specific Stem Cells

**Figure 9:**
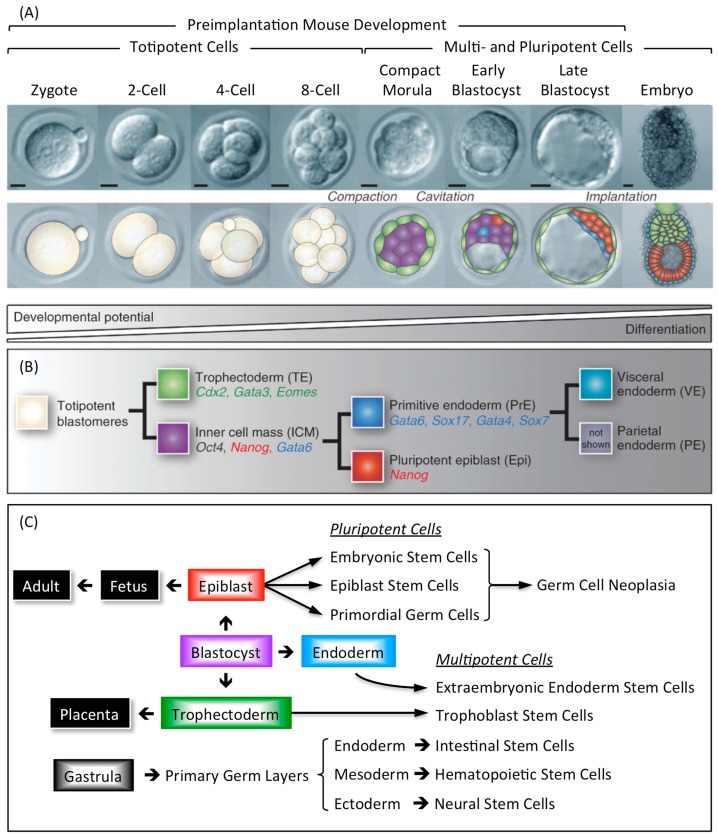
Preimplantation to post-implantation development in the mouse. (**A**) The zygote undergoes three cell cleavage cycles to form an embryo consisting of eight totipotent cells termed blastomeres. The first cell differentiation event in mammalian development begins as totipotent blastomeres become flattened, polar, and are compacted together. During the two following cell cleavage cycles, the outer blastomeres form a monolayer of epithelial cells (the trophectoderm) that envelops the remaining blastomeres (the inner cell mass). Scale bars are 50 μm; (**B**) Proper lineage segregation before implantation is ensured by two cell-fate decisions. The first gives rise to the multipotent trophectoderm and the pluripotent inner cell mass as the totipotent 8-cell embryo develops into a compacted morula. The second leads to the allocation of multipotent primitive endoderm and pluripotent epiblast as early stage blastocyst develops into a late stage blastocyst. After implantation of the embryo, the primitive endoderm differentiates into multipotent visceral and parietal endoderm. Principle biomarkers for various cell types are indicated; (**C**) The origins of pluripotent and multipotent stem cells are indicated. Pluripotent stem cells produce germ cell neoplasias if they migrate to ectopic sites during development, or if they are experimentally transferred to ectopic sites in the fetus or adult. Figure is adapted from [139].

Tissue specific stem cells arise during mammalian development from one of the three primary germ layers that appear upon gastrulation (Figure 9C). The innermost layer is the endoderm, from which is derived the epithelium of the pharynx, respiratory tract, digestive tract, bladder, and urethra. The middle layer is the mesoderm, from which are derived connective tissue, bone, cartilage, muscle, blood and blood vessels, lymphatics, lymphoid organs, notochord, pleura, pericardium, peritoneum, kidneys, and gonads. The outermost layer is the ectoderm, from which is derived the epidermal tissues such as nails, hair, and glands of the skin; the nervous system; external sense organs such as the eye and ear; and the mucous membranes of the mouth and anus. 

Tissue specific stem cells can be either unipotent or multipotent, and they can exist in quiescent or actively dividing states. If a tissue consists of a single cell type, its stem cells are by definition unipotent. Examples are the epidermis, in which basal cells generate only keratinocytes; muscle, in which satellite cells function as unipotent stem cells; and the testis, where spermatocytes are the only cellular output [134]. Hepatocytes could be considered ‘unipotent stem cells’, because they remain quiescent until stimulated to proliferate by physical or chemical damage. Damaging the liver reactivates a ‘neonatal-like’ stem cell program in adult hepatocytes, promoting their proliferation and liver repair, and if the hepatocytes contain an oncogenic mutation, they will produce a liver cancer [15]. 

If a tissue consists of multiple cell types, then its stem cells must be multipotent and have their origins in one of the three germ layers. For example, neural stem cells arise from ectoderm to provide a life-long source of neurons and glia [136], and hematopoietic stem cells that arise from the mesoderm are the source of a complex hierarchical panoply of blood cells [137]. Quiescent hematopoietic stem cells undergo asymmetric cell division during self-renewal to produce actively dividing progenitor cells. As hematopoietic cells differentiate, their repertoire becomes progressively more limited through a series of ordered, irreversible fate decisions to eventually generate the full complement of blood cell types. Tissues such as liver, pancreas, or muscle, display little or no proliferative activity in the adult, but proliferation of their stem cells is activated following tissue damage. In contrast, the endoderm derived intestinal epithelium is one of the most rapidly self-renewing tissues in mammals, because the multipotent stem cells at the base of the crypt proliferate continuously [138].

### 3.2. Embryo Specific Stem Cells

Mammalian development begins when an egg is fertilized by a sperm to produce a 1-cell embryo termed the zygote. The zygote then undergoes preimplantation development to produce a blastocyst that implants into the uterine endothelium during peri-implantation development to produce an embryo (Figure 9A). During mouse development, the 1-cell to 8-cell embryos consist of totipotent blastomeres encapsulated by a thick transparent membrane termed the zona pellucida. During the 8- to 32-cell stage of development, the blastomeres develop cell-to-cell adhesion, and the outer blastomeres differentiate into the multipotent trophectoderm while the remaining blastomeres form the pluripotent inner cell mass. The epithelial trophoblast cells that comprise the trophectoderm give rise only to cells required for implantation and placentation, whereas the inner cell mass gives rise to all of the cell lineages that comprise the embryo, as well as endoderm, mesoderm, and ectoderm components of the placenta [140,141]. The inner cell mass of the blastocyst (recognized by the formation of a blastocoel cavity) differentiates into the pluripotent epiblast and the multipotent primitive endoderm that gives rise to the visceral and parietal endoderm layers following implantation (Figure 9B). 

Stem cells derived from embryos and cultured in vitro can recapitulate all of the developmental changes of their cells of origin when they are transferred to the blastocoel cavity and the blastocyst implanted in a foster mother [142]. Multipotent trophoblast stem cells (TSCs) derived either from preimplantation blastocysts or from the extraembryonic ectoderm of early post-implantation embryos will contribute to the trophectoderm and its derivatives. Similarly, multipotent extraembryonic endoderm stem cells (XENs) derived from the primitive endoderm will give rise to the lineages derived from both visceral and parietal endoderm. Pluripotent embryonic stem cells (ESCs) derived from preimplantation blastocysts and pluripotent epiblast stem cells (EpiSCs) derived from the post-implantation epiblast will give rise to cells derived from all three germ layers (endoderm, mesoderm, and ectoderm) [143,144,145]. Thus, EpiSCs are similar to ESCs, and they can be derived from ESCs [139], except that ESCs represent a more naïve pluripotent state. ESCs can be induced to form TSCs and XENs either by activating or by repressing genes that are critical to either TSC or XEN self-renewal, whereas EpiSCs cannot. Moreover, although EpiSCs can differentiate in vitro and form teratocarcinomas, they have little or no capacity to form blastocyst chimeras when compared with ESCs. 

Primordial germ cells (PGCs) are the immediate precursors for both the male (spermatogonia) and female (oocytes) germ cells [146]. PGCs are specified in the epiblast at the beginning of post-implantation development. They migrate from the epiblast to the genital ridges where they differentiate into either male or female germ cells. However, although PGCs are unipotent in vivo, they reacquire expression of the core pluripotency genes upon gender specification. The core transcriptional regulator proteins that maintain pluripotency (OCT4, SOX2, and NANOG) are first expressed in the inner cell mass and epiblast, but upon epiblast differentiation, SOX2 and NANOG are down-regulated (Figure 9B). As PGCs migrate towards the genital ridges, they continue to express OCT4 and regain the expression of SOX2 and NANOG, thus becoming pluripotent stem cells [146,147]. 

### 3.3. Pluripotent Stem Cells Are Potential Cancer Stem Cells (CSCs)

CSCS and ESCs share many characteristics. Both CSCs and ESCs can differentiate into multiple cell types, and both can retain these properties during self-renewal. Both CSCs and ESCs exhibit rapid proliferation, lack contact inhibition, and express similar genetic signatures [148,149,150,151,152]. ESCs are similar to most cancer cells in that they both operate under low oxygen tension by relying on glycolysis rather than oxidative phosphorylation [153], and they both exhibit genome instability in vitro (particularly human ESCs) [154,155,156]. But most striking is the fact that all pluripotent stem cells, from either mice or humans, produce tumors when inoculated into ectopic sites of isogenic or immuno-compromised fetal or adult mice (ESCs [143,144], EpiSCs [157,158,159], and PGCs [146]).

The tumors produced by pluripotent stem cells resemble closely the spontaneous teratomas and teratocarcinomas that occur early in mouse and human life [160,161,162]. Teratomas are benign tumors that consist of a solid mass of cells haphazardly organized into tissues derived from at least two and usually all three embryonic germ layers. Teratocarcinomas are malignant teratomas from which CSCs, termed ‘embryonal carcinoma cells’ (ECCs), have been isolated. ECCs are remarkably similar to ESCs [145,163], but ECCs have clearly undergone as yet undefined genetic changes that distinguish them from ESCs. Although ECCs can contribute to all tissues of the host embryo, different ECC lines exhibit different properties. Their contributions to embryo development are often limited, they display diverse differentiation properties, and tumors frequently arise in chimeric animals [164,165]. Nevertheless, the ability of pluripotent cells to form extragonadal tumors cannot be duplicated simply by the ubiquitous expression of Oct4 [166]. Accordingly, teratoma formation has been used both as a tool for monitoring pluripotency in stem cell research [162,167] and as a model for embryonic development, disease, and tumorigenesis [168]. Therefore, as development proceeds, pluripotent cells, as exemplified by ESCs, EpiSCs, and PGCs have a demonstrable capability of becoming CSCs should they accidentally find themselves at inappropriate locations.

### 3.4. Take-Home Lesson

Stem cells play a major role both in mammalian development and in maintaining the adult organism. Progenitor cells have been isolated from human cancers that resemble stem cells and therefore are often referred to as CSCs. Whether they are produced naturally during mammalian development or arise in adults remains a matter of intense investigation. CSCs might arise from ESCs that failed to either differentiate or die during fetal development, or they might result from somatic cells in adults that ‘de-differentiated’ in response to environmental stimuli or genetic mutations, thereby returning to a pluripotent state [169]. What is well established is that the pluripotent stem cells that arise during normal development can also produce benign and malignant tumors when located at ectopic sites. Thus, pluripotent stem cells can also function as CSCs.

## 4. Geminin and Germ Cell Neoplasias

Totipotent and pluripotent cells are unique in that Geminin is an essential gene, because Geminin is not essential for the viability of most other cells in adult animals [91,170]. Depletion of Geminin in mouse or human embryonic fibroblasts and in primary human mammary epithelial cells induces senescence instead of DNA re-replication [170,171,172,173], and *Gmnn* ablation in trophoblast stem cells induces terminal differentiation into nonproliferating giant cells [31]. Therefore, Geminin might well be a therapeutic target for cancers that arise from pluripotent cells.

### 4.1. Germ Cell Neoplasias

**Figure 10:**
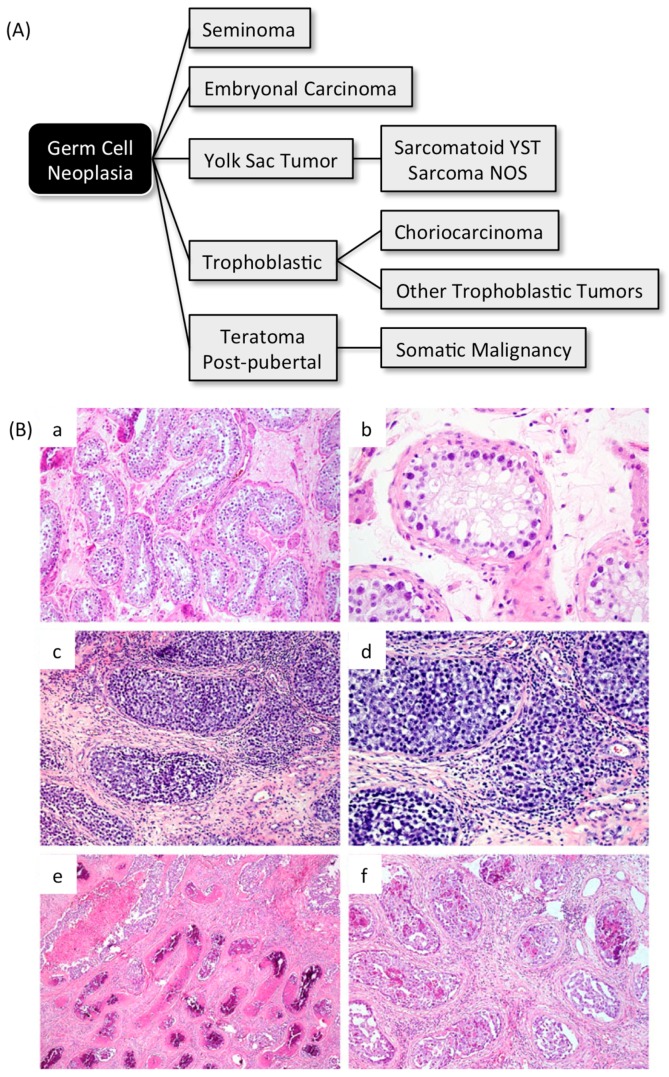
Germ cell neoplasias in situ. (**A**) Germ cell tumor classification is restructured into tumors derived from germ cell neoplasias in situ. (Abbreviations: YST = yolk sac tumor, NOS = not otherwise specified); (**B**) Human germ cell neoplasias in situ typically exhibits an absence of maturing spermatogenesis (**a**) and a conspicuous layer of atypical cells resembling seminoma cells aligned along the basement membrane (the spermatogonial niche, **b**). Intratubular seminoma (**c**) often results in complete filling of seminiferous tubules by seminoma cells, in this example demonstrating both intratubular and invasive components (**d**). Intratubular embryonal carcinoma is characteristically associated with intratubular necrosis and calcification (**e**,**f**). Images and data are from [176].

Teratomas and teratocarcinomas are generic terms for a variety of human tumors termed germ cell neoplasias that originate from pluripotent stem cells (Figure 10A). The progenitors for this form of cancer in humans are presumed to be pluripotent PGCs that originate in the epiblast and then migrate into the endoderm of the umbilical vesicle and via the mesenterium to the genital ridge where gonads eventually form. If PGCs accidentally migrate to ectopic sites, such as the sacro-coccygeal, retro-peritoneal, mediastinal, intracranial, or epiphyseal regions, they form teratomas and teratocarcinomas [174]. However, since the ability to produce teratomas and teratocarcinomas is a characteristic of all pluripotent cells, there is no reason to exclude the possibility that germ cell neoplasias could also arise from other pluripotent stem cells. Some ESCs or EpiSCs, for example, might remain in a quiescent state as development proceeds [175], thereby becoming dispersed among various tissues until environmental signals at ectopic sites trigger their differentiation into teratomas or teratocarcinomas. 

The precursor of adult malignant testicular germ cell tumors is composed of seminoma-like cells with enlarged hyperchromatic nuclei, clumped chromatin, and often prominent nucleoli, aligned along the basement membrane of seminiferous tubules within the spermatogonial niche (Figure 10B, [176]). Similar to seminoma and embryonal carcinoma, these cells are uniformly positive for the embryonic stem cell marker OCT4/POU5F1, and these cells are typical of the embryonal carcinoma cells isolated from experimentally induced teratocarcinomas. Germ cell neoplasias account for about 4% of all childhood tumors [177,178].

Sacrococcygeal teratomas are the most common tumors in newborns, occurring in 1 per 20,000–40,000 births (emedicine.medscape.com, ‘cystic teratomas—epidemiology’). Teratomas of the mediastinum are rare, representing 8% of all tumors of this region. Mature cystic teratomas, the most common ovarian germ cell tumor, account for 10%–20% of all ovarian neoplasms. Testicular cancer is the most common cancer in young men in Western populations, accounting for 1% of all malignancies in men [3]. Germ cell tumors represent 95% of testicular tumors after puberty, but purely benign teratomas of the testis are rare, accounting for only 3%–5% of germ cell tumors.

### 4.2. Geminin Is Essential for Totipotent and Pluripotent Cell Development

Geminin has been reported to have roles both in restricting genome duplication to once per cell division by preventing assembly of prereplication complexes at DNA replication origins during S-phase to mitosis [54,179,180] and in modulating gene expression during cell differentiation [31,181]. Therefore, it is not surprising that Geminin is essential at the beginning of animal development. What is surprising is that Geminin is not essential throughout development.

Geminin depletion in *Xenopus* eggs [182] and *Drosophila* embryos [183] induces genomic instability coincident with the onset of zygotic gene expression, an event that could account for the changes in gene expression observed during the *Xenopus* midblastula transition when Geminin is depleted [184]. Geminin also is essential at the beginning of mouse development. Ablation of Geminin alleles (*Gmnn*) in a mouse zygote results in excess DNA replication and termination of development between the morula and blastocyst stages [170,185,186]. *Gmnn* ablation in newly implanted blastocysts arrests epiblast development [170,187], but the effects of *Gmnn* ablation at later stages in development are less dramatic, suggesting that the importance of Geminin diminishes as development continues [188,189,190]. 

### 4.3. Geminin Prevents DNA Re-Replication Dependent Apoptosis in Pluripotent Cells

What is the role of Geminin in pluripotent cells? Some studies conclude that Geminin is required in preimplantation embryos and ESCs to maintain expression of genes necessary for pluripotency [185,191,192], whereas other studies conclude that Geminin is not required to either maintain or exit pluripotency [170,193], but to prevent aberrant DNA replication from inducing DNA damage and apoptosis [170,186,194]. Paradoxically, these two roles cannot co-exist in the same cell. Otherwise, whenever totipotent and pluripotent cells reduced their Geminin level in order to differentiate, they would trigger DNA re-replication. 

The role of Geminin in ESCs now appears to be resolved. *Gmnn* ablation in ESCs undergoing self-renewal in vitro triggered DNA re-replication followed by DNA damage, a DNA damage response, and then apoptosis [170]. No relationship was detected in these experiments between expression of Geminin and expression of genes associated with either pluripotency or differentiation, and once ESCs differentiated in vitro, they no longer depended on Geminin for viability. To determine whether or not these results were experimental artifacts, immune-deficient mice were inoculated with ESCs containing *Gmnn* alleles that could be ablated by intraperitoneal injections of tamoxifen [91]. If Geminin were essential to maintain pluripotency, then *Gmnn* ablation would stimulate teratoma formation and the resulting tumors would lack *Gmnn* alleles. On the other hand, if Geminin were essential for ESC viability, then *Gmnn* ablation would delay teratoma formation, because most of the ESCs would die and only those that escape *Gmnn* ablation would form teratomas. The results confirmed that Geminin was essential for ESC viability, not for ESC pluripotency. Moreover, once a teratoma was established, the differentiated cells could continue to proliferate in the absence of *Gmnn* alleles, Geminin protein, and pluripotent stem cells. Therefore, Geminin is not essential for viability of differentiated cells in the context of a solid tissue.

The requirement of Geminin for ESC viability in vitro and in vivo accounts for the effects of *Gmnn* ablation in preimplantation embryos. *Gmnn* ablation following fertilization arrested development as embryos entered the morula stage, presumably through depletion of maternally inherited Geminin [170,185,186]. In some cases, the resulting abnormal embryos appeared to be undergoing DNA damage dependent apoptosis [170,186], whereas in other cases they appeared to be undergoing premature differentiation into trophoblast giant cells [185]. A simple explanation would be that the amount of maternally inherited Geminin was greater in the embryos isolated by Gonzalez and co-workers [185] than in the embryos isolated by the Hara [186] and Huang [170] groups. Higher levels of Geminin would allow embryos to develop further before the effects of *Gmnn* ablation were evident. The outer blastomeres would have differentiated into trophoblast cells in those embryos with sufficient Geminin to sustain development to the early morula stage, in which case, depletion of maternally inherited Geminin would kill the remaining totipotent blastomeres while triggering terminal differentiation of the trophoblast cells into giant cells [31].

The role of Geminin in pluripotent cells could also account for the fact that *Gmnn* ablation in the post-implantation epiblast causes neural tube defects through disrupted progenitor specification and neuronal differentiation [187], whereas *Gmnn* ablation in the neural stem cells that appear later during development does not prevent neural development [188,190]. Since the epiblast contains pluripotent progenitor cells from which pluripotent ESCs and EpiSCs are derived [142,157], *Gmnn* ablation in the epiblast would eliminate the pluripotent progenitor cells required to continue development. In contrast, *Gmnn* ablation does not affect either the viability or developmental potential of the multipotent neural stem cells that arise later in development [188], and therefore does not prevent subsequent neural development.

These conclusions are consistent with the fact that Geminin is also required for the mitotic proliferation of undifferentiated male germ cells (spermatogonia) derived from PGCs [195]. *Gmnn* ablation in mouse spermatogonia eliminated them during the initial wave of mitotic proliferation that occurs during the first week of life. Gmnn(-/-) spermatogonia exhibited more double-stranded DNA breaks than control cells, but like ESCs, they maintained expression of genes associated with the undifferentiated state and did not prematurely express genes characteristic of more differentiated spermatogonia. In contrast, *Gmnn* ablation in meiotic spermatocytes did not disrupt meiosis or the differentiation of spermatids into mature sperm. Thus, as with ESCs, Geminin is essential for mitotic proliferation of spermatogonia but not for their differentiation. Therefore, the fact that Geminin is essential for viability, not for regulation of gene expression, in mouse ESCs [91,170] and male germ cells [195] suggests Geminin as a therapeutic target for treatment of human germ cell neoplasias.

### 4.4. Take-Home Lesson

Evidence is accumulating that pluripotent stem cells also reside among adult tissues, where they maintain their ability to differentiate into multiple types of tissue-specific stem cells [196]. If these pluripotent cells can also produce tumors and require Geminin for viability, then Geminin might well be a chemotherapeutic target for many types of CSCs. In fact, depletion of Geminin in 23 different human cell lines revealed that Geminin was essential to prevent DNA re-replication in cells derived from six different cancers, but it was not essential in all cancer cells, and not in cells derived from normal tissues [54,86,98,179]. Cells that were insensitive to depletion of Geminin were sensitive to depletion of both Geminin and Cyclin A, consistent with the existence of multiple concerted pathways to prevent DNA re-replication (Figure 7). Ironically, overexpression of Geminin in human mammary epithelial cells promotes tumor formation in immune-compromised mice [90], underscoring the fact that the relationship between protein levels is critical. 

## 5. Conclusions

Both the accumulation of genetic mutations and the induction of unscheduled genome duplication could initiate adult cancers, but it is the ability of DNA re-replication and unscheduled genome duplication to induce polyploidy and aneuploidy that provides cancer cells with extra copies of genes, thereby allowing these cells to become more aggressive and to resist chemotherapy. Simply put, genome instability is advantageous to the formation and survival of adult cancers. Whether or not these characteristics also apply to germ cell cancers remains to be explored. Although at least 42 genes have now been identified that are essential for preventing excess DNA replication in at least one form of cancer, identifying which of these genes is selectively required in cancer cells, but not normal cells, opens the door to a new strategy for cancer selective therapy: targeting a gene that prevents genome stability with its accompanying DNA damage, together with a second gene that is essential to repair DNA damage. Geminin is but one example of such a target. Future studies need to target Geminin in CSCs derived from adult tissues, and need to determine which, if any, of the other genes that are essential for preventing excess genome duplication exhibit broad based selectivity for cancer cells compared to normal tissues.

## Figures and Tables

**Table 1:** Genes Essential to Prevent Excess Genome Duplication in HCT116 cells [55].

Gene	Function
**Origin Licensing Block**	
FBXO5/Emi1	inhibits APC/C
GMNN/Geminin	inhibits Cdt1
CUL1/Cullin 1	CRL1 E3-ubiquitin ligase subunit
NEDD8	
RBX1/ROC1	
DTL/Cdt2/DCAF2	CRL4 E3-ubiquitin ligase subunit
DDB1	
**Chromatin Untangling**	
TOP2A/Topoisomerase IIα	resolves catenated intertwines
**Mitotic Entry & Maintenance**	
LIN54	regulates G2→M transition
CCNB1/Cyclin B1	initiates and maintains mitosis
MASTL/Greatwall	accelerates entry into mitosis and blocks exit from mitosis
PLK1/Polo-like kinase 1	mitotic entry, centrosome maturation, microtubule nucleation
SMC2	condensin subunits, chromosome condensation during mitosis
SMC4	
**Mitotic Spindle Assembly**	
TPX2	promotes spindle assembly
KIF11/Eg5/Kinesin-11	required for bipolar spindle formation
CEP192	required for centriole duplication
AURKA/Aurora kinase A	builds bipolar spindle, regulates centrosome separation and microtubule dynamics
POC1A/WDR51A	ensures centriole integrity
**Spindle Assembly Checkpoint**	
INCENP	Chromosome Passenger Complex (CPC)
BIRC5/Survivin	
CDCA8/Borealin	
AURKB/Aurora kinase B	
CASC5/D40/KNL1	KMN network component, ensures MCC assembly
BUB3	recruits SAC proteins to kinetochore
BUB1B	Mitotic Checkpoint Complex (MCC)
MAD2L1/MAD2	
TTK/Mps1	stimulates CPC and MCC
NUF2	NDC80 kinetochore complex subunit
**Sister Chromatid Cohesion**	
CDCA5/Sororin	inhibits cohesin dissociation
PPP2R1A/PP2A-alpha	prevents cohesin phosphorylation
SGOL1/Sgo1/Shugoshin-like 1	targets PPA2 to centromeric cohesin
**Chromosome Segregation**	
ESPL1/Separase	cleaves cohesin
CDC16/APC6	Anaphase Promoting Complex (APC/C)
CDC26/APC12	
CDC27/APC3	
**Cytokinesis**	
ANLN/Anillin	crosslinks filaments in contractile ring
PRC1	midzone formation
RACGAP1	Centralspindlin
ECT2	
KIF23/MKLP1/Kinesin-23	
CHMP4B	component of the ESCRTIII complex

**Table 2:** Genes essential to prevent aneuploidy and tumors in mice [55].

Genes	Cell Cycle Event	*Aneuploidy	*Tumors	Ref.
PLK1/ Polo-like Kinase 1	Mitotic Entry & Maintenance	Yes	Yes	[104]
TPX2	Mitotic Spindle Assembly	Yes	Yes	[105]
KIF11/Eg5/Kinesin-11		Yes	Yes	[106]
AURKA/Aurora Kinase A		Yes	Yes	[107]
INCENP/ Inner Centromere Protein	Spindle Assembly Checkpoint	Yes		[108,109]
BIRC5/Survivin		Yes		[109]
CDCA8/Borealin		Yes		[110]
AURKB/Aurora Kinase B		Yes		[111]
BUB3		Yes		[112,113]
BUB1B		Yes		[114]
MAD2L1/MAD2/ Mitotic Arrest Deficient		Yes	Yes	[115,116,117,118]
TTK/Mps1		Yes	Yes	[119]
SGOL1/Sgo1/ Shugoshin-like 1	Sister Chromatid Cohesion	Yes	Yes	[120]
ESPL1/Separase	Chromosome Segregation	Yes	Yes	[121]

* Mouse models expressing either haplo-insufficient or hypomorphic alleles in which the gene is under-expressed. Aneuploidy is defined simply as an abnormal number of chromosomes in a fraction of the cells statistically greater than controls, and polyploidy is defined simply as an excess number of chromosomes. Although the majority of cells contained 40 chromosomes (diploid), some cells contained as few as 36 chromosomes and others contained as many as 80 chromosomes [105,112,114].
